# Roughness assessment of colored compomers: Results after an erosive-abrasive in vitro cycling test

**DOI:** 10.4317/jced.60544

**Published:** 2023-06-01

**Authors:** Louise Magalhães, Fernanda-Vieira Belém, Kamilla França, Cristiane-Meira Assunção, Paulo-Antônio Martins-Júnior, Ana-Paula Turrioni, Marco-Aurélio-Benini Paschoal

**Affiliations:** 1 Undergraduate student, Federal University of Minas Gerais – UFMG, Dental School, Belo Horizonte, Minas Gerais, Brazil; 2 DDS, Post Graduate Program in Dentistry, Federal University of Minas Gerais – UFMG, Dental School, Belo Horizonte, Minas Gerais, Brazil; 3 Undergraduate student, Federal University of Uberlândia, – UFU, Dental School, Uberlândia, Minas Gerais, Brazil; 4 DDS, MSc, PhD, Adjunct Professor, Department of Child and Adolescent Oral Health, Federal University of Minas Gerais – UFMG, Dental School, Belo Horizonte, Minas Gerais, Brazil; 5 DDS, MSc, PhD, Federal University of Uberlândia, – UFU, Dental School, Uberlândia, Minas Gerais, Brazil

## Abstract

**Background:**

The present *in vitro* study aimed to evaluate and compare the surface roughness of a colored compomer and a composite resin, after 15 days of erosive-abrasive cycling.

**Material and Methods:**

The sample included ninety circular specimens, randomly divided (n = 10): G1 Berry, G2 Gold, G3 Pink, G4 Lemon, G5 Blue, G6 Silver, G7 Orange and G8 Green, referring to the different colors of compomer (Twinky Star®, VOCO, Germany) and G9 for composite resin (Z250®, 3M ESPE). The specimens were submerged in artificial saliva and stored at 37°C for 24 hours. After polishing and finishing, the specimens were submitted to initial roughness (R1). Then, the specimens were submerged in an acidic cola-based drink for 1 minute and then exposed to electric toothbrushing for 2 minutes for 15 days. After this period, the final roughness (R2) and the ΔRa were performed. Data were submitted to ANOVA and Tukey’s test for intergroup comparison and paired T-test for intragroup comparison (*p*<0.05).

**Results:**

Among compomers, the green color presented the higher/lower initial and final roughness values (0.94 ± 0.44, 1.35 ± 0.55) with lemon color presenting the most prominent real roughness increase (ΔRa = 0,74) whereas composite resin showed the lower values (0,17 ± 0.06, 0,31 ± 0.15; ΔRa = 0,14).

**Conclusions:**

All compomers, after the erosive-abrasive challenge, presented an increase in roughness values when compared to composite resin with a highlight to green tones.

** Key words:**Compomers, composite resins, surface properties.

## Introduction

Compomer is a hybrid restorative material that presents in its structure aesthetic and mechanical properties of resin composites (80%), associated with the fluoride release ability of glass ionomer cement (20%) (1-5). A compomer containing different color shades was developed to perform restorations in deciduous posterior teeth, aiming to offer a more attractive material for children and help in the management of clinical behavior (4,5).

Unlike conventional compomers, this material is available in some colors with amount of glitter particles incorporated into its structure, resulting in a shiny effect during the brushing process (1,4,5). Despite the interesting proposal, considering the possible stimulus to oral hygiene regarding to the uniqueness of the presence of diversified colors and the scintillating effect, few studies focusing on its characterization are available. Thus, it is unknown how these new characteristics can affect the physical properties of dental restorations and this aspect represents a point of attention for dental professionals, especially pediatric dentists (1,4,5).

Roughness property is directly linked to the adhesion of substrates to the surface of dental materials and teeth, due to the alteration of the surface smoothness, which is represented by undercut areas. Clinically, higher values of roughness can increase the biofilm deposition which can generate marginal infiltrations, caries recurrence and even periodontal alterations, reducing the restoration survival (6,7).

Erosion and dental abrasion represent some variables directly linked to the increase in surface modifications of dental materials. Exposure to acids found in commercially available beverages and food, associated with mechanical efforts during tooth brushing, can cause an increase in surface roughness (8,9,10,11,12,13).

There are few studies evaluating the association of these factors with this specific type of dental material. Hence, this present investigation aimed to investigate and compare the surface roughness of this colored compomer and a conventional composite resin, after an *in vitro* erosive-abrasive cycling.

## Material and Methods

-Tested materials

The present study used the following materials: colored compomer (Twinky Star®, VOCO, Germany) and composite resin (FiltekZ250®, 3M ESPE, USA). The sample size calculation was defined based on the pilot study and the parameters used were surface roughness of the colored compomer and conventional composite resin, after an erosion/abrasion cycling, assuming 5% significance level and 80% study power, considering sample loss of 10%, reaching a value of n = 10, for each of the tested groups.

-Specimen preparation

The specimens were prepared in a 10 mm diameter x 2mm depth circular acrylic matrix and protected with a polyester matrix. After compression with a glass slide and the sample was light cured (14). In addition, the materials were handled according to the manufacturer’s guidelines by a properly trained operator.

The specimens were divided as described in Table 1 and stored in artificial saliva at 37º, for 24 hours. Subsequently, finishing and polishing were performed with sandpaper discs (#400, #600, #1200, #1500, #2000) using a metallographic polisher, with pressure control aiming to sample standardization (5).

**Table 1:**
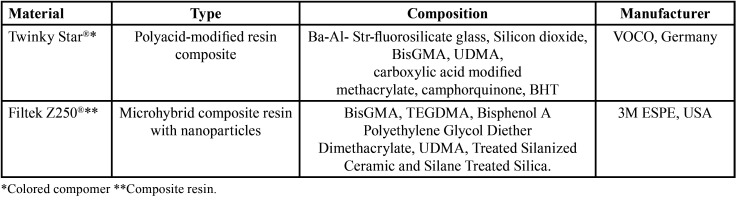
Materials specifications.

-Mechanical property characterization

The roughness measurement (µm) was performed using a roughness meter (Mitutoyo Corporation, Japan) following ISO 1997 (cut-off at 0.8 and 0.5 mm/s). In this process, the average was taken by arithmetic of 5 readings, respecting the whole test surface, for each specimen.

-Erosive/abrasive cycling test

For the erosive/abrasive cycling test, all specimens were exposed to acidic coca-based solution (Coca Cola®) for 1 minute and then simulated brushing was performed using an electric toothbrush (Oral-B Professional Care 5000®) with fluoride toothpaste (Colgate Total 12®) for 2 minutes, during 15 days (19). Before and after this procedure the samples were submitted to roughness measurement considering R1 and R2 for initial and final roughness measurements, respectively. Accordingly, the real increase in roughness (ΔRa = R2 – R1) was calculated accordingly.

-Statistical analysis

The data were submitted to the normality Kolmogorov-Smirnov test. After this, one-way ANOVA test, followed by the post-hoc Tukey test were used for intergroup evaluation. In addition, the paired T-test was applied to verify the intragroup materials comparison. For all tests, the *p* value was considered at 5% using statistical software package SPSS for Windows (SPSS, version 24.0, IBM Corp, Chicago, IL, USA).

## Results

The comparison between initial and final roughness values after 15 days of erosive-abrasive cycling is presented in Table 2.

**Table 2:**
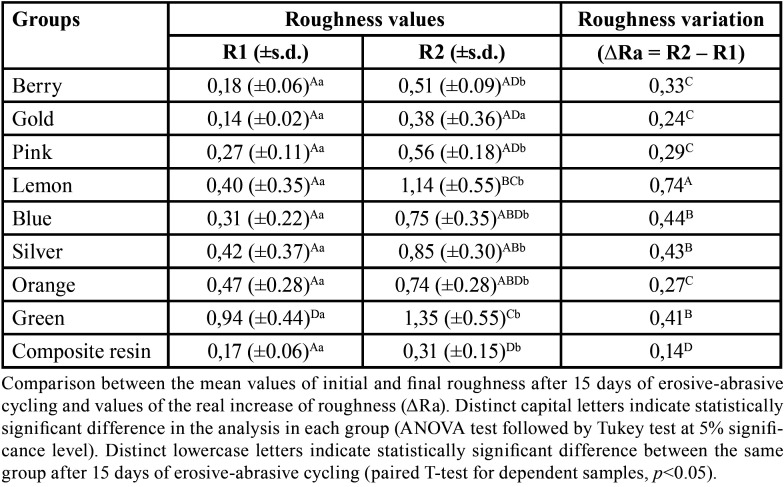
Mean values of initial roughness and after 15 days of erosive- abrasive-cycling and the real increase of roughness values (ΔRa).

As verified, the green compomer presented higher initial and final roughness values (0.94 ± 0.44; 1.35 ± 0.55). In summary, all studied materials showed an increase of roughness after the erosive/abrasive cycling test. Still, composite resin showed the lowest ΔRa value (0.14) whereas the gold compomer color presented the closest value (ΔRa = 0.24).

## Discussion

This study revealed that composite resin showed the lowest alteration in surface roughness of the specimens and the green shades of studied colored compomers (green and lemon) reached the highest roughness values after the erosive/abrasive cycling test (1.35 ± 0.55 and 1.14 ± 0.55, respectively).

Similarly, Bors *et al*. (15) verified that composite resin presented higher depth loss compared to Twinky Star® (VOCO, Germany) compomer analyzing the behavior of restorative materials in erosive conditions study. Different degradation after exposure to low pH drink can be a result of the dissimilar chemical composition and structure of these restorative materials.

However, these findings contrast with another study in the literature (16), which found no significant increase on roughness means for same both studied materials The storage using other product including citric acid after submersion in acidic cola-based drink and the storage period possibly influenced this different outcome.

The color of restorative materials receives the influence of the type and content of the pigment in the material (17). In contrast to conventional compomer or composite resin, the Twinky Star® (VOCO, Germany) colored compomer exhibits 8 different pigments and shimmering particles incorporated into its structure (3,4). These glitter particles are included in order to reach colour effect shades (16,17). The results of this study demonstrated that the green shades increased final roughness and the differences between the surface roughness values can be associated to the organic or inorganic pigments. Furthermore, it is believed that roughness variation may be associated with the composition of the materials, colors, and glitter amount (9-12).

On the other hand, the gold colored compomer presented the closest roughness variation value compared to composite resin, followed by orange and pink shades (ΔRa = 0.24, 0.27, 0.29). Specifically, for this colored material, light absorbance and reflectance and the curing protocol may be responsible for these values, since the color may reflect the number of glittering particles and light transmission as well.

The present *in vitro* study has a limitation because it was carried out in a laboratory environment with controlled variables. The characteristics of this colored material highlight some questions regarding its performance in the oral environment, including the property of surface roughness. The literature reports (8) that superficial changes in restorative materials contribute directly to greater retention of biofilm and influence on the decrease of restoration longevity.

Since the surface roughness of restorations can be affected by internal and mainly external factors, the investigation of the interaction of variables such as erosion and dental abrasion with the colored compomer roughness it is a valid study purpose. Extrinsic erosion caused by the ingestion of commercially available beverages with low pH and dental abrasion by toothbrushing forces are factors that can significantly affect the surface of dental materials, especially when there is an association between both (8).

The challenge with the cola-based acid drink was chosen considering its indiscriminate widespread consumption by children (9). This habit requires more attention by the whole population, considering that the ingestion of acidic beverages may result in local (e.g., dental and restorations) and general (e.g., obesity, vascular diseases) health issues (13,18). For the dental abrasion test, electric toothbrushes with soft bristles covering the entire area of the specimen were chosen, in addition to the good performance in removing biofilm previously reported in findings in the literature (9,19,20).

The production of new studies related to the physicochemical characterization and evaluation of the clinical performance of these materials should be carried out, for a better understanding of their use in dentistry.

## Conclusions

It was observed that the groups referring to the green tones (green and lemon), were the ones that stood out the most in relation to the increase in roughness when submitted to this erosive-abrasive cycling. It is strongly recommending further investigations focusing on in situ and *in vivo* approaches aiming to better elucidate its performance and use of this compomer in pediatric dental daily practice.
